# Empirical Logic for Bio-Inspired Soft Computing: Illustrative Applications in Control Engineering and Cluster Analysis

**DOI:** 10.3390/biomimetics11060437

**Published:** 2026-06-18

**Authors:** Jens Grotrian

**Affiliations:** Chair of Business Administration, in Particular Planning, Innovation and Founding, Brandenburg University of Technology Cottbus-Senftenberg, Erich-Weinert-Straße 1, D-03046 Cottbus, Germany; jens.grotrian@posteo.de

**Keywords:** empirical logic, soft computing, approximate reasoning, bio-inspired computing, nature-inspired computing, control engineering, cluster analysis

## Abstract

Empirical logic (EL) is a bio-inspired soft computing approach to rule-based decision-making that emphasizes intuitive, experience-based reasoning. While its theoretical foundations have been established in previous work, its practical applicability and accessibility have so far received less attention. This paper addresses this gap by providing two representative application examples from distinct domains: control engineering and cluster analysis. The first example demonstrates the use of EL for the speed control of a DC drive, highlighting its ability to achieve competitive dynamic performance with a small number of intuitive rules. The second example introduces a novel approach to cluster analysis, where cluster structures emerge from the collective interaction of EL rules rather than from the optimization of a predefined objective function. In addition, the paper emphasizes the availability of publicly accessible software realizations of EL, including a Maple-based prototype and a Python framework, which enable direct experimentation and practical use. By combining illustrative applications with executable tools, the paper aims to facilitate the transition from conceptual understanding to practical deployment and to support further exploration of EL in applied soft computing contexts.

## 1. Introduction

Cognitive processes in living organisms can exhibit a remarkable ability to handle vague concepts, uncertain situations, incomplete information, and context-dependent knowledge in a fast and adaptive manner. In contrast to strictly formal reasoning, such processes are often based on experience, qualitative assessment, and the integration of partially valid information.

Empirical logic (EL) is a rule-based framework motivated by these principles. It is designed to process vague or partially reliable knowledge using graded validity instead of binary truth values. Within a neuron-inspired category network, hypothesis validities emerge from the simultaneous interaction of indication-based excitatory and inhibitory rules.

The theoretical foundations of EL, including its operators and validity concepts, have been developed in detail in previous work by the author, particularly in the article *Thinking fast: a bionic approach to soft computing* [1] and the accompanying doctoral thesis, where the theoretical foundations and core concepts of EL are presented in detail [2] (pp. 29–32).

Rather than further elaborating on these foundations, the present contribution focuses on the practical applicability of EL. In particular, it complements the previous theoretical exposition by demonstrating how EL can be effectively applied in concrete problem domains and made accessible to practitioners. To illustrate the approach, the paper examines two representative applications from distinct fields—control engineering and cluster analysis—highlighting the modeling principles, interpretability, and flexibility of EL in practice.

To support practical adoption, publicly accessible software realizations of EL have been developed, including a Maple-based prototype [3] and the Python framework *empirical-logic 1.0.1* [4]. These tools are intended to facilitate the transition from conceptual understanding to practical use and to encourage further exploration of EL in bio-inspired reasoning and soft computing contexts.

The following two sections are intended to remind the reader of the bio-inspired approach and principles of EL.

### 1.1. The Biological Motivation of Empirical Logic (EL)

*Empirical logic* is an innovative, rule-based soft computing approach for decision-making grounded in expert knowledge. As a heuristic method that does not claim the rigor of a formal logical system, it is especially well suited to handling vague concepts. Its development is grounded in a bionic perspective, drawing inspiration from the functioning of the human subconscious, referred to in psychology and neuroscience as *System 1*.

System 1 represents the fast and largely automatic ability of the human brain to instinctively categorize perceptions by associating them with emotional reactions (e.g., fear) and stored experiences. Its efficiency stems from the parallel processing of sensory information within biological neural networks.

This capability contrasts with that of *System 2*, which, together with System 1, forms the basis of the so-called “dual-process theory” as described in *Thinking, Fast and Slow* by Daniel Kahneman (Nobel Prize in Economic Sciences 2002) [5,6,7]. System 2 refers to the deliberate cognitive processing of perceptions triggered by System 1 and presented to conscious awareness for evaluation before an action or decision is made. Owing to its sequential nature, System 2 operates significantly more slowly.

While System 2 is particularly characteristic of more recently evolved species such as humans, System 1 is fundamentally linked to the earliest forms of life. This aspect has received comparatively less attention than higher-level cognitive functions in modern artificial intelligence (AI) research. In contrast, the development of EL follows the premise that investigating the origins of cognition in simple life forms may yield more efficient computational principles.

As early as 1987, David D. Tank and John J. Hopfield (Nobel Prize in Physics 2024) [8] published results from experiments with neuron-like electronic circuits that support the view that the collective excitation propagation of System 1 structurally resembles emergent activation dynamics in biological neural networks. In their article, the two neuroscientists report that electronic circuits based on neurobiological models are able to solve complex problems rapidly. They emphasize that their particular interest in such artificial neural circuits lies in understanding the operating principles of cognitive perception processes as a result of self-optimization (i.e., *emergence*) of collectively interacting system components.

Using the Maple computer algebra system (CAS) [9] (p. 998ff), the collective processing effects described by Tank and Hopfield were successfully replicated. For this purpose, a prototype of an EL software library was developed and tested using several practical application examples from business administration, economics, operations research and control engineering [3].

### 1.2. Some Principles of Empirical Logic (EL)

The basic mechanism of a nervous system consists of the directed transmission of signals of varying intensity between neurons. Synapses play a key role in determining whether the activity of a receiving neuron is excited or inhibited [10,11].

This communication principle is reflected in the fundamental concepts of EL:

1. Notions used to organize perceived information from our environment, such as facts, individuals, observed characteristics, actions, decisions, etc., are abstracted by EL into the concept of a *category* corresponding to a neuron.
2. Each category has a dynamic state, referred to as *validity*, defined on a bipolar interval from −1 to +1. A value of +1 (−1) indicates that a fact fully belongs to (does not belong to) the category.
3. Categories may be either *bound* or *free*, depending on whether they are linked to a scalable reference magnitude.
4. EL defines four standardized validity functions that suffice to characterize any bound category by means of location and shape parameters (e.g., *fuzziness*):
  - Threshold function (*e_THRESHOLD*).
  - Limitation function (*e_LIMITATION*).
  - Interval function (*e_INTERVAL*).
  - Estimation function (*e_ESTIMATION*).
5. Each validity function has a corresponding inverse function (e.g., *e_THRESHOLD_INVERSE*).
6. The semantic foundation of EL is based on the *paradigm of circumstantial evidence*, where both *hypotheses* and supporting or opposing indications (*pro-* or *contra-indications*) are represented as categories.
7. Asserting a category validity (e.g., approving a hypothesis) is represented by the *e_VALIDATE* operator and corresponds to an *excitatory synapse*.
8. Refuting a category validity (e.g., disproving a hypothesis) is represented by the *e_INVALIDATE* operator and corresponds to an *inhibitory synapse*.
9. *e_AND*, *e_OR*, and *e_NOT* form a functionally complete set of logical operators used to construct the premise argument for *e_VALIDATE* and *e_INVALIDATE*.

For the computational replication of collective processing effects observed in biological neural networks, it proved crucial that the operators were implemented using the functions listed in Table 1 and that the applied C- and D-norms fulfill the *neutrality axiom*:

(1) $$min\left\{x_{1},\ldots ,x_{n}\right\}\leq C\left(x_{1},\ldots ,x_{n}\right)\leq max\left\{x_{1},\ldots ,x_{n}\right\}$$

(2) $$min\left\{x_{1},\ldots ,x_{n}\right\}\leq D\left(x_{1},\ldots ,x_{n}\right)\leq max\left\{x_{1},\ldots ,x_{n}\right\}$$

The neutrality axiom of C- and D-norms postulates that a logical combination of statements cannot be a source of (previously unavailable) information. This means that the result of a logical combination must not indicate a higher degree of certainty than is warranted by the degrees of certainty of the individual statements. In other words, a logical combination should not be ampliative [14] (p. 125), [15].

The relevant mathematical details can be found in [2] (pp. 68–90) and [1] (pp. 8–13).

## 2. Speed Control of a DC Drive Using Empirical Logic (Application Example)

**Figure 1:**
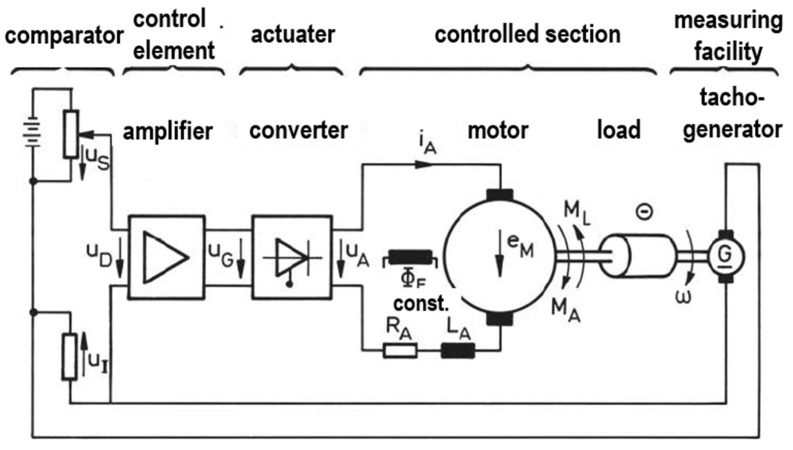
Speed control of a DC drive (image reproduction courtesy of VDE-Verlag).

The following example of the speed control of a DC drive is taken from a well-known textbook on control engineering [16], and its implementation with EL can be found in more detail in the doctoral thesis of the author [2] (pp. 183–200). The control element labeled “amplifier” in Figure 1 is to be replaced and examined below by suitably dimensioned controllers of the PI, PID and EL (“EL” here refers to an empirical logic control unit) types.

**Figure 2:**
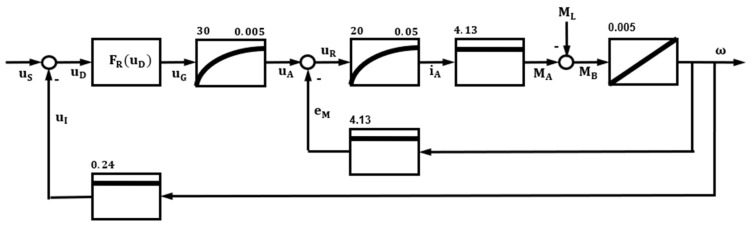
Block diagram of the DC drive speed control loop (for the definitions of the symbols used in the block diagram, refer to Table 2-1 in [16] (p. 45)).

For the structural analysis and the derivation of the equations that describe the electrical behavior of the motor, the mechanical movement of the motor armature with load, and the transfer behavior of the converter circuit and the control amplifier, see Föllinger [16] (pp. 90–93). The results of a functional analysis are reflected in the structural picture of the control circuit shown in Figure 2.

### 2.1. Definition of Control-Relevant Categories

The relevant reference magnitudes for the yet-to-be-determined bound categories of an EL controller can be read from Figure 2, as follows (the neutral designations e(t) instead of $u_{D}\left(t\right)$ and u(t) instead of $u_{G}\left(t\right)$ correspond to the nomenclature customary in control engineering):

  $$\begin{array}{c} e\left(t\right)=\text{deviation}\;u_{D}\;\text{of}\;\text{the}\;\text{feedback}\;\text{variable}\;u_{I},\;\text{which}\;\text{is}\;\text{proportional}\;\text{to}\;\text{the}\;\text{controlled}\;\text{variable}\;\omega ,\;\text{from}\;\text{the}\;\text{reference}\;\text{variable}\;u_{S}\;\text{at}\;\text{the}\;\text{controller}\;\text{input}\\ u\left(t\right)=\text{correcting}\;\text{variable}\;u_{G}\;\text{at}\;\text{the}\;\text{controller}\;\text{output}\; \end{array}$$

In addition, it is useful to compute the change (first derivative) of the controlled variable deviation to be used as a further reference magnitude:

(3) $$c\left(t_{n+1}\right)=\frac{e\left(t_{n+1}\right)-e\left(t_{n}\right)}{∆t}$$

∆t denotes the duration between two consecutive sampling times $t_{n}$ and $t_{n+1}$ (the simulation results explained in Section 2.3 were obtained with ∆t=0.0001).

Since, after a closer look at the inner loop of the block diagram of Figure 2, the DC drive turns out to be a controlled section with three time constants, stabilization of the control loop requires taking into account the acceleration (second derivative) of the controlled variable deviation e(t) as a fourth reference magnitude for the design of the EL controller (the corresponding analysis with an equivalent transformation of the block diagram can be found in Föllinger [16] (pp. 90–93)):

(4) $$a\left(t_{n+1}\right)=\frac{c\left(t_{n+1}\right)-c\left(t_{n}\right)}{∆t}$$

**Figure 3:**
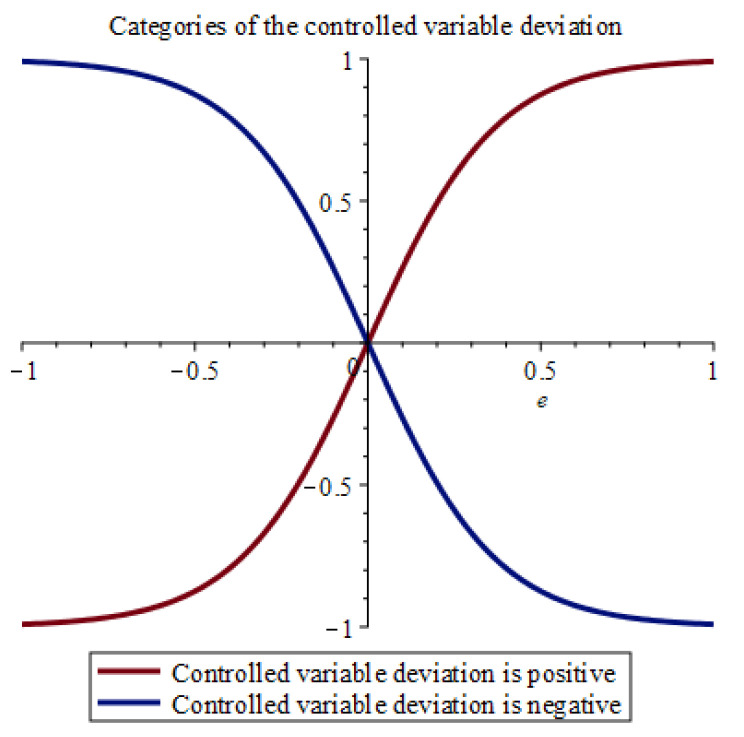
Bound categories belonging to reference magnitude “Controlled variable deviation e(t)” [V].

**Figure 4:**
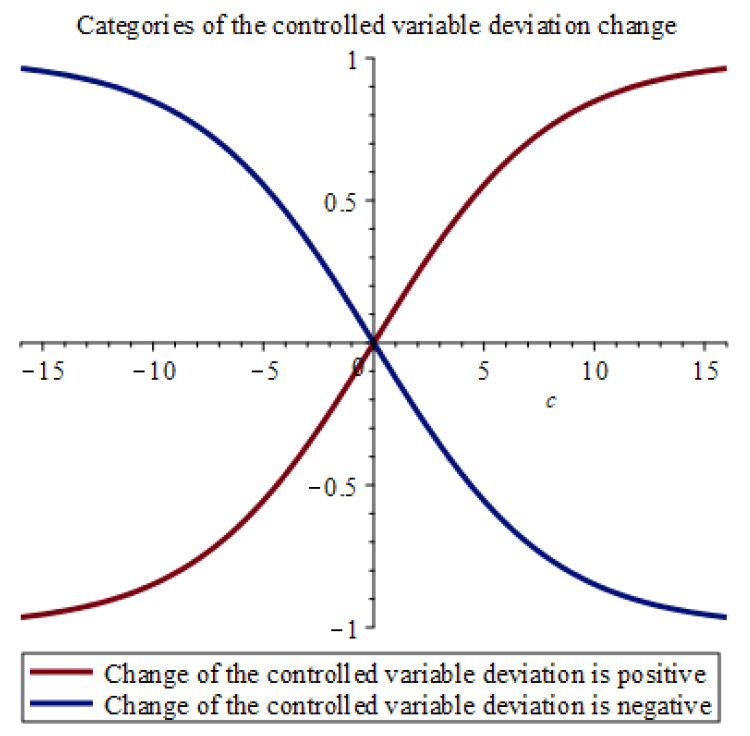
Bound categories belonging to reference magnitude “Change of the controlled variable deviation c(t)” [V/s].

**Figure 5:**
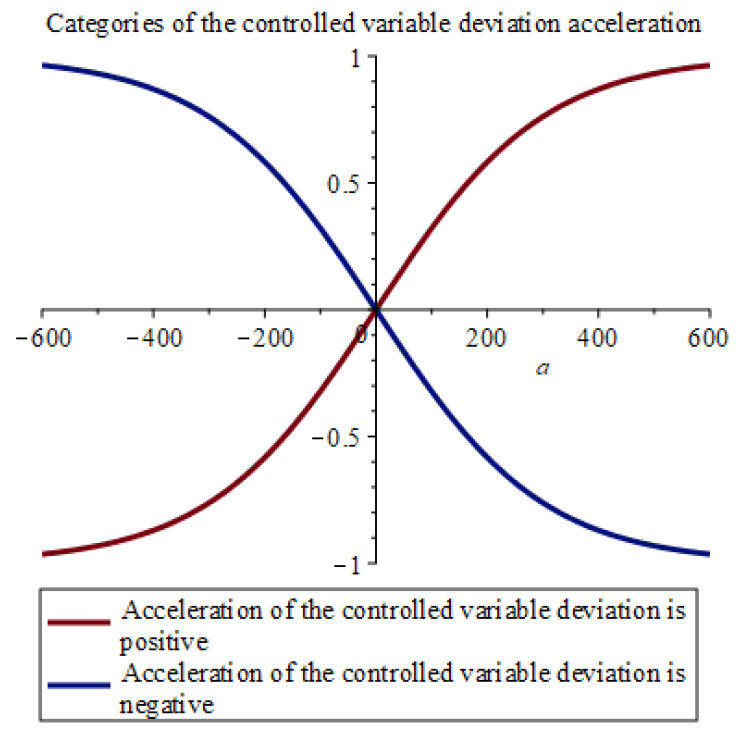
Bound categories belonging to reference magnitude “Acceleration of the controlled variable deviation a(t)” [V/s^2^].

**Figure 6:**
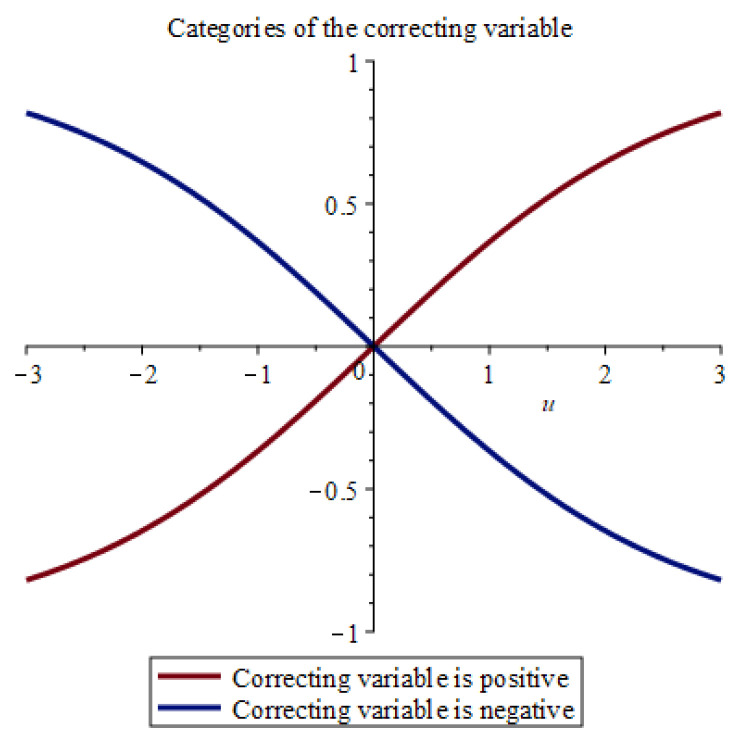
Bound categories belonging to reference magnitude “Correcting variable u(t)” [V].

The bound categories shown in Figure 3, Figure 4, Figure 5 and Figure 6 are derived from the reference magnitudes mentioned above (for the sake of simplicity, the reference values are denoted as dimensionless).

Among the available validity functions, the threshold function *e_THRESHOLD* and the limitation function *e_LIMITATION* are suitable for representing these categories. Table 2 shows these with the corresponding arguments assigned. The rationale for the specific selection of the fuzziness parameter values is given below (see Section 2.3).

### 2.2. The Empirical Logic Rules of the DC Drive Speed Control

The only two rules required for controlling a DC drive with the help of EL can be expressed in such a way that they correspond to intuition and do not require any additional explanation (Table 3):

For the rule conclusions, the inverse validity functions e_THRESHOLD_INVERSE and e_LIMITATION_INVERSE are used, such that (the syntactic details of these functions can be found in [2] (p. 40ff) or in the Python source code of the empirical logic 1.0.1 framework [4]):

(5) $$Increase\_the\_correcting\_variable∶=\{\begin{array}{ccc} V & \to & \left[0,\;1\right]\\ u & \mapsto & e\_THRESHOLD\_INVERSE\left(u,-5,\;5,\;0,\;2.6\right) \end{array}$$

(6) $$Decrease\_the\_correcting\_variable∶=\{\begin{array}{ccc} V & \to & \left[0,\;1\right]\\ u & \mapsto & e\_LIMITATION\_INVERSE\left(u,-5,\;5,\;0,\;2.6\right) \end{array}$$

**Figure 7:**
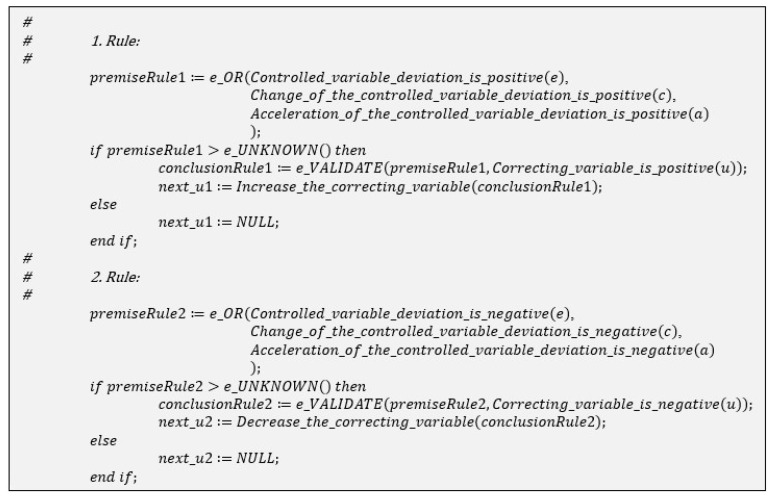
The EL rules are translated into Maple code.

Figure 7 shows the translation of the rules presented in Table 3 into Maple code.

**Figure 8:**
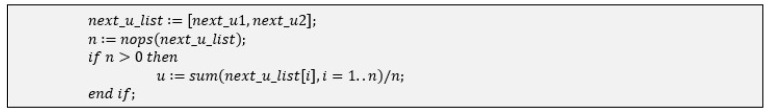
Resolving a rule conflict.

In principle, the premises of both rules may be satisfied simultaneously, leading to competing new settings for the manipulated variables *next_u1* and *next_u2*. In this case, it must be specified how the resulting value *next_u* of the control variable *u* is derived from the two proposed values. A simple solution is to take their arithmetic mean (Figure 8):

### 2.3. Simulating the Speed Control Using Empirical Logic

The EL controller was tested using a Maple-based simulation environment, allowing comparison of its dynamic behavior with that of optimally tuned PI and PID controllers [16] (p. 180ff). The design of a PI or PID controller for the DC drive from Figure 1 can be found in Föllinger [16] (pp. 185–188). Established synthesis methods and rules of thumb exist for conventional controller types. However, due to its nonlinearity, these methods are not applicable to EL controllers. Therefore, simulation experiments are required to determine the controller parameters. Here, “controller parameters” refers to the *fuzziness* parameter of the validity functions for the bound categories of the DC drive speed control system (see Figure 3, Figure 4, Figure 5 and Figure 6). However, it is possible to take advantage of the effect of different degrees of fuzziness of the categories based on the results of the rule applications. Rules respond sensitively to low fuzziness and more smoothly to higher fuzziness.

As a rule of thumb, the following guideline applies:

1. Category fuzziness of the reference magnitude at the controller input $\approx \frac{1}{4}$ span of the value range.
2. Category fuzziness of the reference magnitude at the controller output $\approx \frac{1}{2}$ span of the value range.

When using a control system developed with EL for the first time, the value range can be difficult to assess. However, for the DC drive speed control, it is feasible to adopt the value ranges from the simulations with a PI or PID controller and, if necessary, to experimentally adjust the fuzziness parameters derived from them. A PI controller with a loop gain of V = 50, for example, yielded the values listed in Table 4.

**Figure 9:**
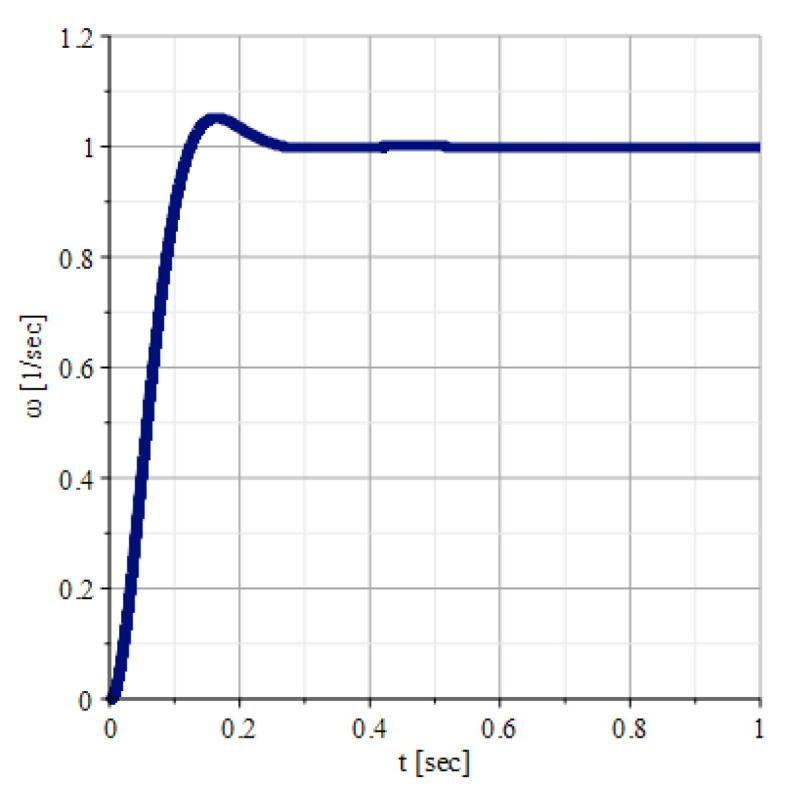
Reference step response of the EL controller.

**Figure 10:**
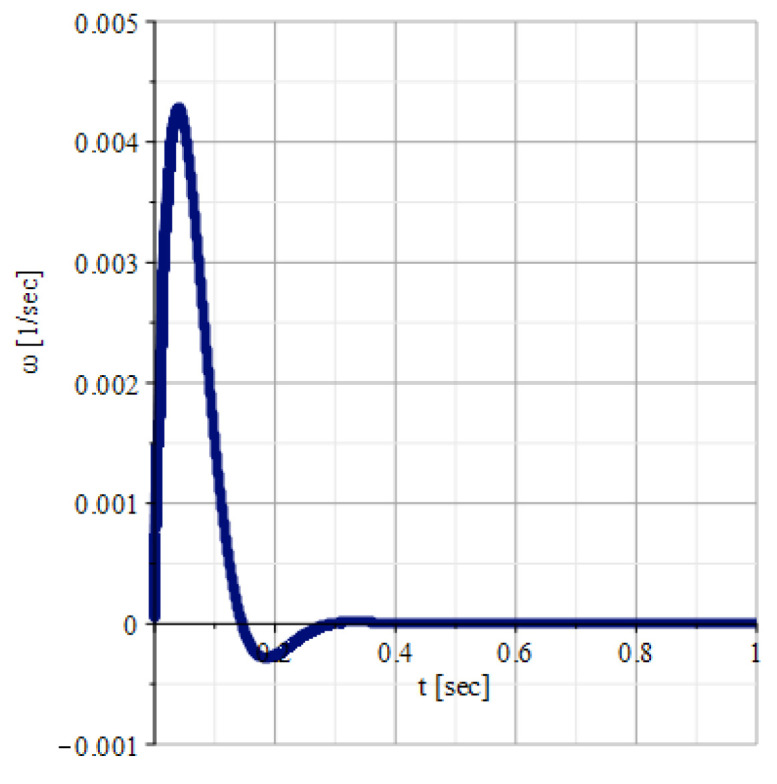
Disturbance step response of the EL controller.

With these parameter settings, the reference and disturbance step responses of the DC drive operated with an EL controller are shown in Figure 9 and Figure 10.

### 2.4. Comparing the Simulation Results

**Figure 11:**
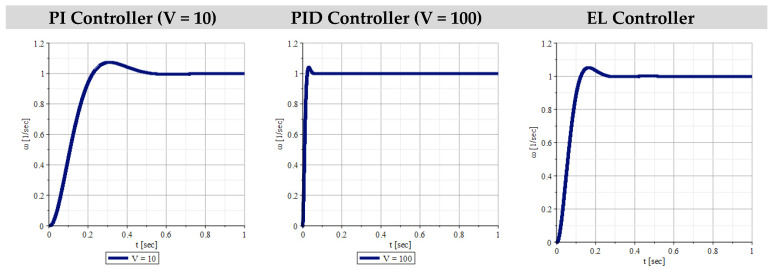
Comparing the reference step responses.

**Figure 12:**
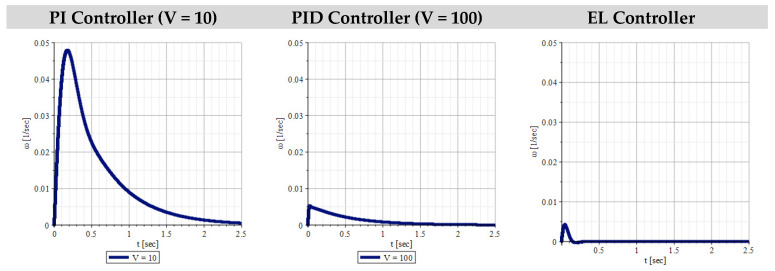
Comparing the disturbance step responses.

The graphs of the step responses shown in Figure 9 and Figure 10 use different scales in order to be able to display the curves as precisely as possible. However, a uniform scale is required for a direct comparison of the different controller types. In addition, a loop gain V should be chosen such that optimal stabilization behavior is achieved for the respective controller type. As shown in Figure 11, the EL controller responds to a reference step change faster than the PI controller but slower than the PID controller. After a disturbance step, the controlled variable deviates only slightly from the setpoint—like the PID controller—and reaches the steady-state condition even faster (see Figure 12).

## 3. Cluster Analysis (Application Example)

Cluster analysis is a multivariate method in exploratory data analysis. It is used to uncover intrinsic structures in large data sets by grouping classification objects that are similar based on their characteristics, such as companies, products, buyers, etc., into so-called clusters [17], ([18] p. 78ff), [19,20]. This method is particularly useful for highly heterogeneous data sets, so that common statistical parameters such as mean or variance are of little significance as indicators for the entire survey. Clustering therefore attempts to partition the data set into groups with greater homogeneity (intragroup homogeneity) [21]. Using an intentionally simple economic question, the following sections show that EL is well suited to this task.

### 3.1. A Simple Application Example

**Figure 13:**
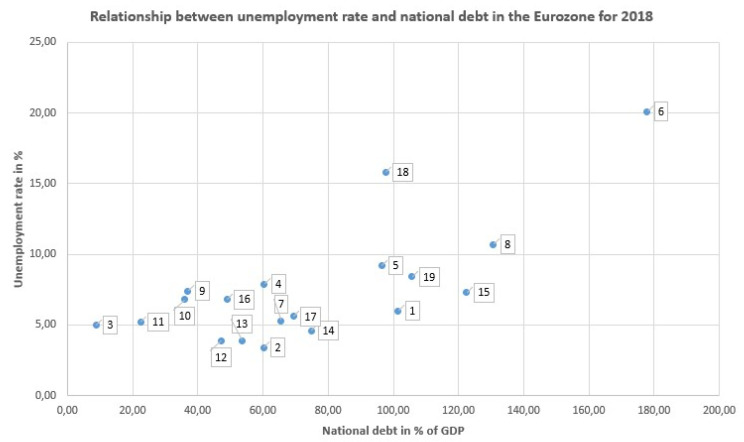
Data set of the application example (scatter plot).

The application example concerns the relationship between unemployment and national debt in the Eurozone for the year 2018 [22]. The set of classification objects to be partitioned into clusters therefore consists of the 19 countries of the European Union with the euro currency (EU-19) (see Table 5 and Figure 13). Assuming that each country can serve as a potential cluster center (i.e., *cluster reference object*), cluster analysis can be formulated as a combinatorial assignment problem between the EU-19 countries and up to 19 cluster reference objects.

If the assignment decisions are denoted by $x_{ij}\in \left\{0,\;1\right\}$, the problem does not represent a one-to-one assignment; rather, several of the m=19 countries i may be assigned to the same cluster j:

(7) $$\sum_{i=1}^{m}x_{ij}\leq m$$

Conversely, each country i must belong to exactly one cluster j:

(8) $$\sum_{j=1}^{m}x_{ij}=1$$

### 3.2. The Pursued Approach

The approach pursued here is based on the following conceptual model: according to *Coulomb’s law* of electrostatics, it is well known that two unlike charges $Q_{1}$ and $Q_{2}$ attract each other with a force F that is inversely proportional to their distance *r* [23] (pp. 19–27), [24,25]:

(9) $$F=\frac{Q_{1}Q_{2}}{4\pi \varepsilon r^{2}}$$

Analogously, it is assumed that the classification objects in a cluster analysis task also exert mutual attractive forces on each other that are inversely proportional to their distance measure. Accordingly, the attractive force of a cluster reference object increases with the number of “neighboring” or “similar” classification objects. It is therefore assumed that classification objects grouped within a cluster do not exhibit a “small distance” by chance, but that this arrangement is a causal consequence of a hypothetical attractive force associated with the cluster reference object (see Section 3.5.2 below).

Based on the conceptual system introduced by the pioneering Dutch computer scientist Gerrit Blaauw [1], the following sections first present the functional appearance of cluster analysis from the user’s perspective (the *system architecture*) and then the internal functional system structure (the *system implementation*). However, a detailed explanation of the *system realization* as a prototype using the computer algebra system Maple is intentionally omitted [2] (pp. 149–162; 353–360).

### 3.3. Architecture of the Cluster Analysis System

The software of a cluster analysis program implemented with EL requires at least three inputs:

1. Data set of classification objects.
2. Parameter controlling the strength of the attractive force of the cluster reference objects.
3. Maximum number of EL rule iterations if no feasible solution is found.

The data set is typically represented as a table:

(10) $$Dataset={\left({attr}_{ij}\right)}_{m,n}\in R^{m\times n}$$

**Figure 14:**
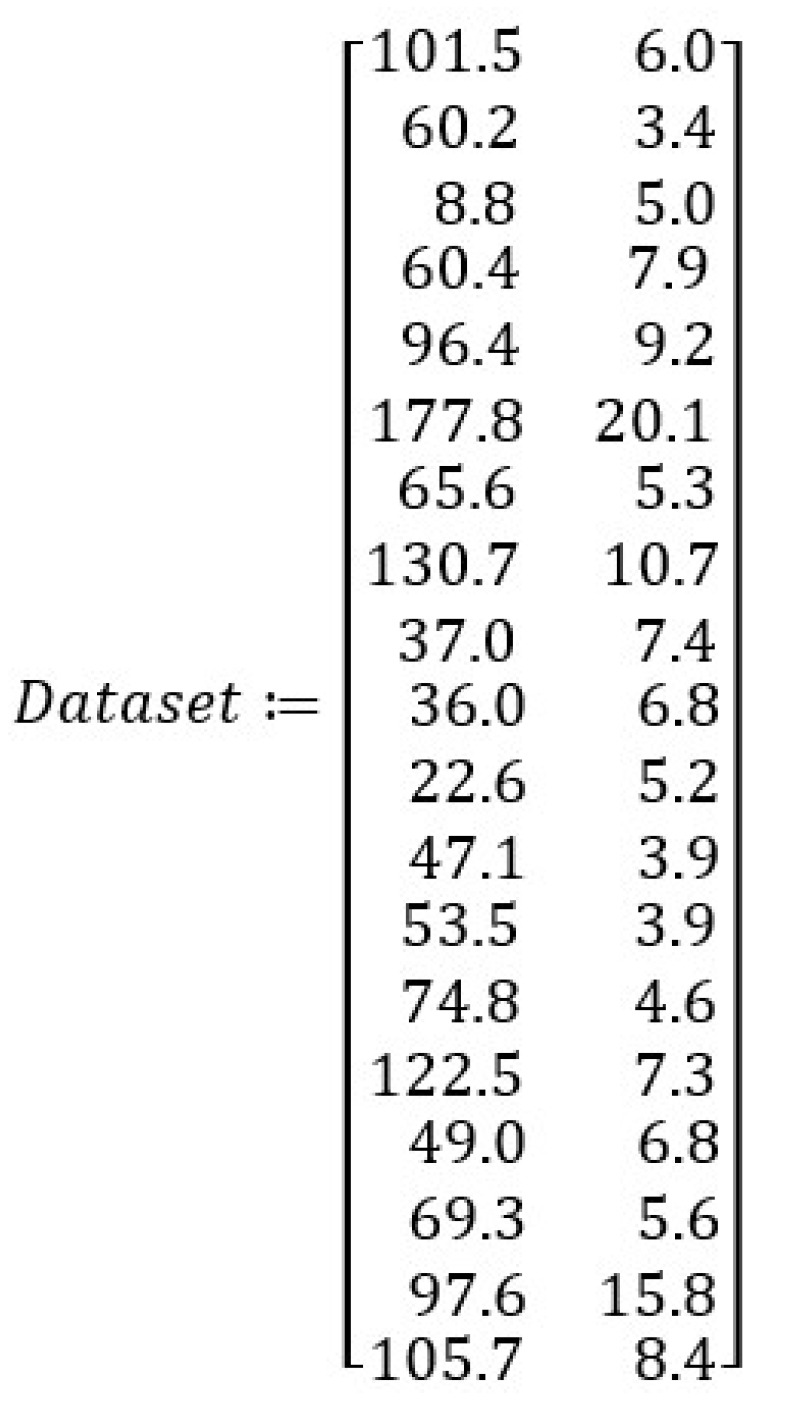
Data matrix.

This is conveniently represented as a matrix such that each row contains the attribute values of the n characteristic features for m classification objects. For the example involving the 19 European Union countries using the euro, the data matrix is shown in Figure 14:

A user-defined parameter, *CARC* (*Cluster Attraction Range Coefficient*), determines the strength of the attractive force exerted by cluster reference objects on neighboring or similar classification objects. It is defined in the interval [0, 1], corresponding to the range between the minimum and maximum attractive force. In combination with another user-defined parameter specifying the maximum number of rule iterations, the Maple-based prototype is invoked as follows:

(11) ExecuteClusterAnalysis(Dataset, CARC, maxIterations)

The program execution result consists of three elements, including a return code RC, the number of required rule executions, and a cluster assignment matrix. The respective meanings of the return codes are listed in Table 6.

Note that the return code RC=−2 means that with a large value of the parameter maxIterations, a possibly futile search for a valid cluster partitioning was aborted prematurely, since no change in the validity values of the cluster assignment matrix is detectable upon further execution of the EL rules, and the system is apparently close to a stationary equilibrium.

**Figure 15:**
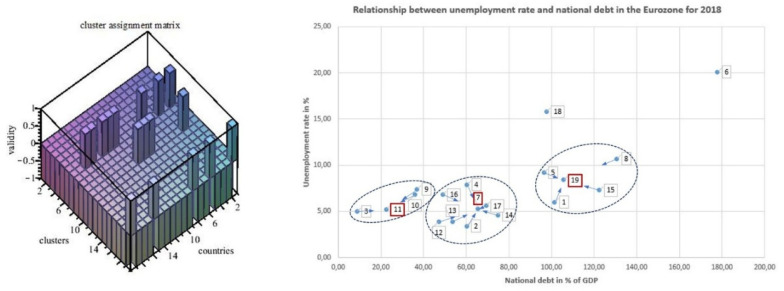
Result of a cluster analysis with CARC = 0.3.

**Figure 16:**
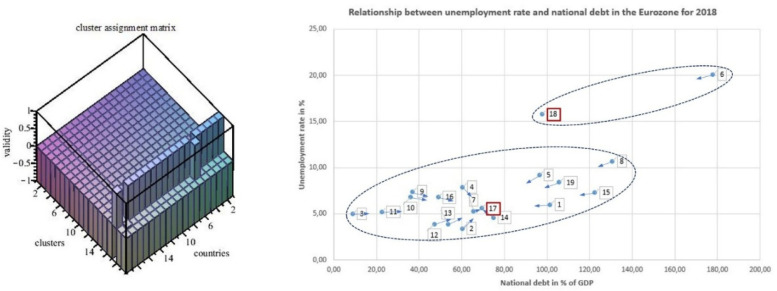
Result of a cluster analysis with CARC = 0.6.

**Figure 17:**
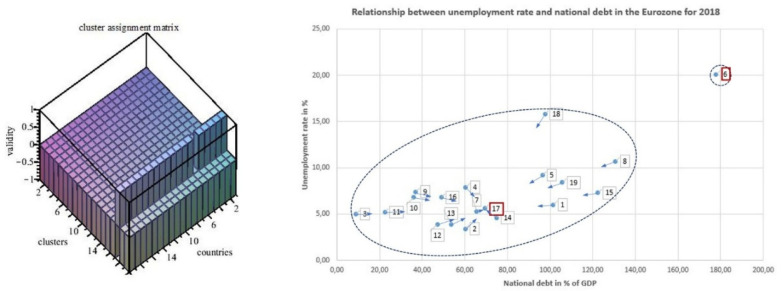
Result of a cluster analysis with CARC = 0.9.

**Figure 18:**
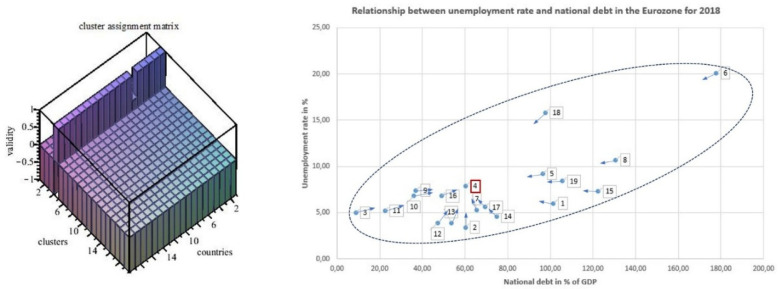
Result of a cluster analysis with CARC = 1.0.

The returned cluster assignment matrix $D={\left(d_{ij}\right)}_{m,m}\in V^{m\times m}$ is quadratic. For each classification object i, it indicates the validity of the assignment $d_{ij}\in V$ to the cluster reference object j. Figure 15, Figure 16, Figure 17 and Figure 18 graphically show the cluster assignment matrix as a bar chart for several different values of the parameter CARC. The corresponding clusters are marked with a dashed line in the data set shown as a scatter plot. The cluster reference object located in the center of each cluster is highlighted in red. Arrows indicate the directions of the respective attractive forces.

Note that the result of the cluster analysis for CARC=0.3 (see Figure 15) is formally invalid according to the criteria mentioned in Section 3.1, since countries 6 (Greece) and 18 (Spain) could not be assigned to any cluster. Although the program terminated with return code RC=−2 (see Table 6) after reaching a steady state, the partitioning into three clusters and two “special cases” clearly provides the most meaningful basis for a more detailed fiscal and employment policy assessment of the year 2018 compared to the other cluster configurations (see Figure 16, Figure 17 and Figure 18). The absence of Greece and Spain from any cluster can be attributed to the fact that, with the parameter value CARC = 0.3, the two classification criteria—unemployment rate and national debt—differ too strongly from those of the other Eurozone countries to allow the formation of a homogeneous group. A positive attractive force can only be observed once the *CARC* parameter value increases further, either acting on these two countries or emanating from them as cluster reference objects.

Additionally, it should be noted that the positive validity achieved for cluster reference object 6 (Greece) in Figure 17 is too small to be visible in the left-hand bar chart.

### 3.4. Implementation of the Cluster Analysis System

For the implementation of the cluster analysis system prototype, the internal structure of another EL application developed for solving the “book sorting problem” was reused and adapted to the specific requirements of the task (see Chapter 4 in the above-mentioned paper by the author *Thinking fast: a bionic approach to soft computing* [1] (pp. 16–18)). For a better understanding of the following sections, it may be useful to first study the explanations given there.

The implementation of the cluster analysis system architecture outlined in Section 3.3 consists of the following procedural steps:

1. Determine a *feature distance matrix* for each classification feature.
2. Determine a *categorized feature matrix* for each feature distance matrix by applying the validity function of the bound category “small distance”.
3. Combine the categorized feature distance matrices into a *categorized data set matrix* using the EL operator *e_AND*.
4. Determine the attractive force value for each potential cluster reference object.
5. Interpret the attractive force values as validity values of the free category “great attractive force”.
6. Turn the categorized data set matrix into an *assignment preference matrix* by replacing its diagonal elements with the validity values of the category “great attractive force”.
7. Apply the cluster analysis empirical rules to the assignment preference matrix.

The details of these operations are discussed in the following sections.

### 3.5. The Categories of Assignment Preferences

#### 3.5.1. The Category “Small Distance”

According to step 1 of the procedure described above, a symmetric feature distance matrix

(12) $${\left(\Delta_{ij}^{k}\right)}_{m,m}\in R^{m\times m}$$

must be determined for each of the k=1 … n columns of the data set matrix. Their entries represent the reference magnitudes for the category “small distance”. These are defined on the set of m classification objects for the feature k by the relation

(13) $$P_{k}={\left(p_{ij}^{k}\right)}_{m,m}\in V^{m\times m}\mathrm{with}\;p_{ij}^{k}=e\_LIMITATION(\Delta_{ij}^{k},l_{k},f_{k})$$

It corresponds to the statement:

“Classification object i has a small distance to classification object j with respect to feature k.”

The parameters $l_{k}$ (=validity limit) and $f_{k}$ (=fuzziness) required for the limiting function *e_LIMITATION* are calculated for each k according to the following formulas:

(14) $$l_{k}=\frac{CARC\cdot {max}_{i\neq j}\left(\Delta_{ij}^{k}\right)+\left(2-CARC\right)\cdot {min}_{i\neq j}\left(\Delta_{ij}^{k}\right)}{2}$$

(15) $$f_{k}=max\left(CARC\cdot \frac{{max}_{i\neq j}\left(\Delta_{ij}^{k}\right)-{min}_{i\neq j}\left(\Delta_{ij}^{k}\right)}{6},f_{min}\right)\mathrm{with}\;f_{min}>0$$

The minimum fuzziness value $f_{min}$ avoids the invalid case $f_{k}=0$  (e.g., $f_{min}=0.001$).

**Figure 19:**
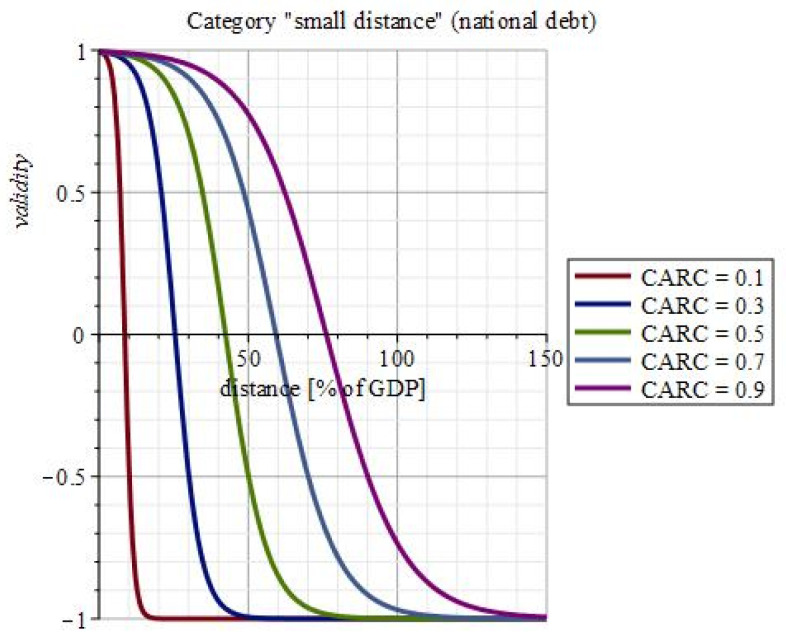
Validity function graphs of the bound category “small distance” (national debt).

**Figure 20:**
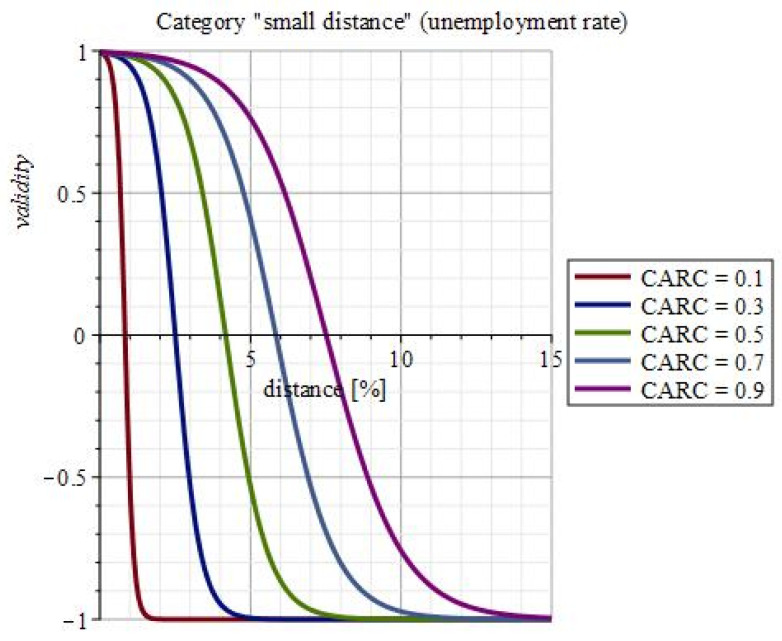
Validity function graphs of the bound category “small distance” (unemployment rate).

Figure 19 and Figure 20 show that a change in the range of attractive forces of the cluster reference objects, controlled by the *CARC* parameter, results in a corresponding parallel shift in the validity limit $l_{k}$ and an adjustment of the fuzziness $f_{k}$ in the validity function graph of the “small distance” category.

**Figure 21:**
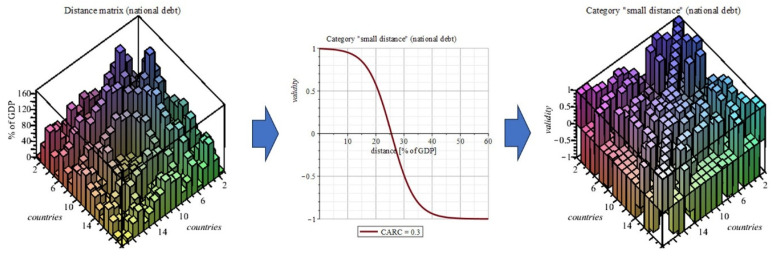
Determine “small distances” between national debts.

**Figure 22:**
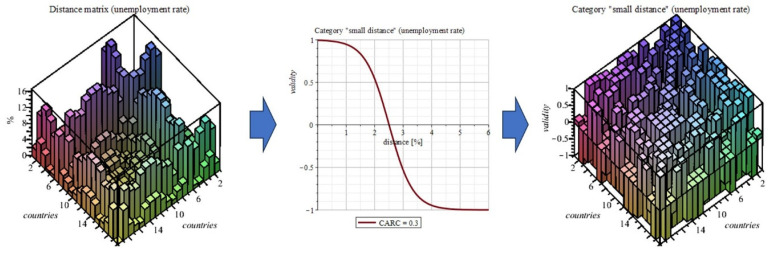
Determine “small distances” between unemployment rates.

Figure 21 and Figure 22 illustrate, in the form of bar charts, how the categorized feature matrices are derived from the distance matrices using the validity function of the bound category “small distance”, with CARC = 0.3 as an example (see step 2 of the implementation procedure).

**Figure 23:**
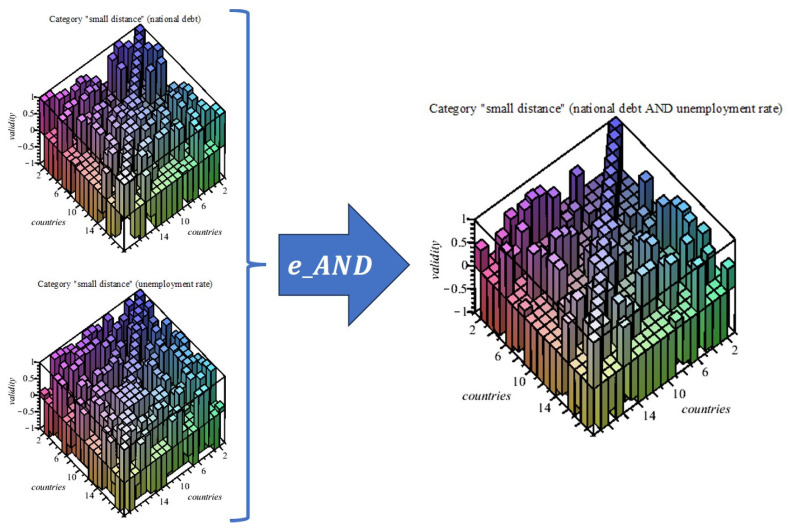
Determine “small distances” between classification objects (Eurozone countries).

Figure 23 shows how the EL operator *e_AND* serves to combine the k=1 … n categorized feature matrices (relations $P_{k}$) into a general relation on the set of m classification objects as an n-fold bound category (see implementation step 3):

(16) $$P={\left(p_{ij}\right)}_{m,m}\in V^{m\times m}$$

The resulting categorized data set matrix assesses the validity of the statement

“Classification object i has a small distance to classification object j.”

An equivalent formulation is: “The classification objects i and j are similar”.

#### 3.5.2. The Category “Great Attractive Force”

Figure 23 also indicates that the diagonal elements $p_{ii}$ of the category “small distance,” always hold a validity value 1=e_TRUE, since each classification object has zero distance to itself. This implies that, due to their lack of distinguishability, they have no meaningful relevance for the empirical rules of cluster analysis described below in Section 3.5.4. However, this deficiency can be remedied by replacing the diagonal elements with the validity values of another assignment preference, with the meaning “great attractive force”. As a free category, this expresses, for each classification object, the extent to which it is suitable to serve as a “power center” or as a cluster reference object that binds classification objects at a “small distance” as cluster elements.

**Figure 24:**
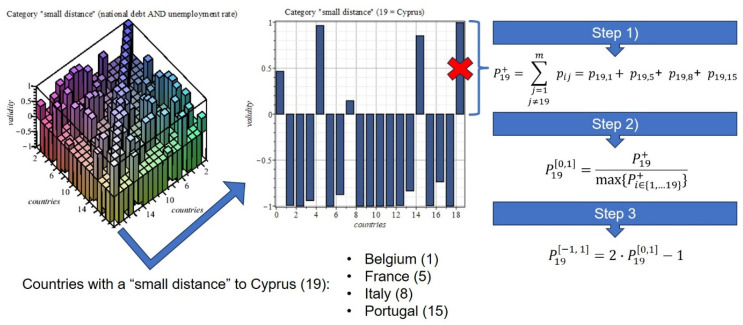
Calculating the validity values of category “great attractive force”.

**Figure 25:**
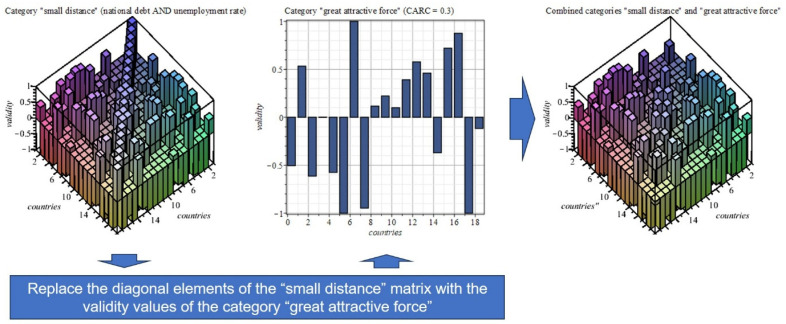
Determine the assignment preference matrix.

The following procedure has proven useful for calculating the corresponding validity values (see Figure 24 and Figure 25, and steps 4 to 6 of the implementation procedure):

(1) For each row i of the matrix $P={\left(p_{ij}\right)}_{m,m}\in V^{m\times m}$, determine the sum of the validity values with $p_{ij}>0$ without considering the diagonal element $p_{ii}$:

(17) $$P_{i}^{+}=\sum_{\begin{array}{c} j=1\\ j\neq i\; \end{array}}^{m}{\;p}_{ij}\;\text{with}\;p_{ij}>e\_UNKNOWN()=0$$

(2) Scale the determined validity sums to the validity interval [0,1]:

(18) $$P_{i}^{\left[0,\;1\right]}=\frac{P_{i}^{+}}{max\{P_{i}^{+}\}}\;\text{for}\;i=1\;\ldots \;m$$

(3) Transform the scaled validity sums to the interval [−1, 1] using the mapping R: [0, 1]→ [−1, 1] and R(v)=2v−1:

(19) $$P_{i}^{\left[-1,1\right]}=R\left(P_{i}^{\left[0,\;1\right]}\right)\;\text{for}\;i=1\;\ldots \;m$$

(4) Replace the diagonal elements of the matrix P by the validity values calculated in step 3) with the new meaning:

“Classification object i has a great attractive force as a cluster reference object.”

#### 3.5.3. The Category Assignment Decisions

**Figure 26:**
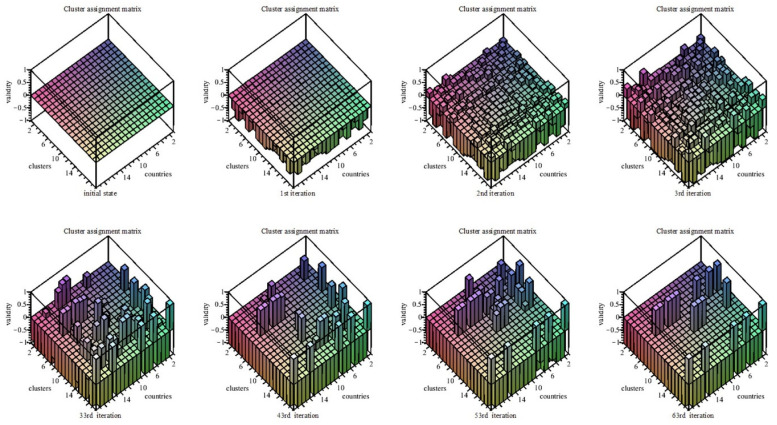
Validity of assignment decisions during the execution of the cluster analysis.

The cluster assignment matrix $D={\left(d_{ij}\right)}_{m,m}\in V^{m\times m}$, already mentioned in Section 1.2, represents—as a free category (see Section 1.2)—the totality of assignment decisions between the classification objects and those classification objects selected as cluster reference objects during the cluster analysis. In the initial state, these have the validity value *e_UNKNOWN*, i.e., the value zero. During cluster analysis, the iterative application of the empirical rules described below induces seemingly chaotic, non-monotonic, and non-linear fluctuations in the validity values until the termination criterion in Section 3.5.5 is met (see bar charts in Figure 26). This demonstrates that the collective interactions involved in rule execution give rise to the spontaneous formation of ordered structures, commonly referred to as *emergence*.

#### 3.5.4. The Empirical Rules of Cluster Analysis

Unlike conventional cluster analysis methods, the empirical rules do not prescribe an algorithmic solution strategy but instead define the properties of a “permissible” cluster partitioning, which is easier to obtain. Due to the assignment preferences “small distance” and “great attractive force” encoded in the assignment preference matrix *P* (see Section 3.5.2), the rules must account for the corresponding case distinctions (see Table 7 and Table 8).

The formal description of the rules using the EL operators for the diagonal elements i = 1…m of the assignment preference matrix $P={\left(p_{ij}\right)}_{m,m}\in V^{m\times m}$ and the cluster assignment matrix $D={\left(d_{ij}\right)}_{m,m}\in V^{m\times m}$ with $i\notin \left\{k_{1},k_{2},\;..,k_{m-1}\right\}$ is as follows (the first argument of *e_(IN)VALIDATE* denotes the rule premise, and the second argument denotes the rule conclusion):

(20) $$d_{ii}^{t+1}≔e\_VALIDATE(e\_AND(p_{ii},\;e\_NOT(d_{ik_{1}}^{t},\;d_{ik_{2}}^{t},\;\ldots ,\;d_{ik_{m-1}}^{t})),\;d_{ii}^{t})$$

(21) $$d_{ii}^{t+1}≔e\_INVALIDATE(e\_OR(e\_NOT(p_{ii}),\;d_{ik_{1}}^{t},\;d_{ik_{2}}^{t},\;\ldots ,\;d_{ik_{m-1}}^{t}),\;d_{ii}^{t})$$

Here, $d_{ii}^{t}$ and $d_{ii}^{t+1}$ denote the validity values of the assignment decisions at the times before and after the execution of the rules.

The corresponding formalization of the rules for the off-diagonal elements of the two matrices has this appearance:

(22) $$d_{ij}^{t+1}≔e\_VALIDATE(e\_AND(p_{ij},p_{jj},\;e\_NOT(d_{ik_{1}}^{t},\;d_{ik_{2}}^{t},\;\ldots ,\;d_{ik_{m-1}}^{t})),\;d_{ij}^{t})$$

(23) $$d_{ij}^{t+1}≔e\_INVALIDATE(e\_OR(e\_NOT(p_{ij}),e\_NOT(p_{jj}),\;d_{ik_{1}}^{t},\;d_{ik_{2}}^{t},\;\ldots ,\;d_{ik_{m-1}}^{t}),\;d_{ij}^{t})$$

The entirety of these validation and invalidation rules forms a network of collectively interacting assignment decisions. However, certain precautions must be taken when implementing these rules to ensure that the principle of collective interaction is correctly reflected:

(1) It is both permissible and advantageous to apply all validation rules first, followed by all invalidation rules, to the assignment decisions $d_{ij}$ in each iteration phase *t*.

(2) However, the rules must not be executed on the same cluster assignment matrix $D^{t}={\left(d_{ij}^{t}\right)}_{m,m}\in V^{m\times m}$, since the resulting validity values of the assignment decisions would otherwise depend on the order of execution and thus be distorted. Therefore, it is necessary to explicitly introduce a second matrix data structure $D^{t+1}={\left(d_{ij}^{t+1}\right)}_{m,m}\in V^{m\times m}$, which stores the results computed in iteration *t*. In iteration t+1, the two data structures exchange their roles, a process commonly referred to as *swapping* in software terminology.

#### 3.5.5. The Termination Criterion for the Rule Iterations

The application of the empirical rules of cluster analysis has a certain similarity to a *production system*. This term refers to a well-known architecture of knowledge-based systems, the functional principles of which are described in great detail, for example, in [26]. However, there are some essential differences:

The EL rules are always executed collectively (i.e., virtually “simultaneously”), whereas in a production system the set of selectable rules (the so-called *conflict set*) is recomputed at each iteration and only one rule is executed. While the inference engine of a production system terminates once the conflict set no longer contains any applicable rules, the control flow of the EL rules requires an explicit termination criterion. Otherwise, their application would not terminate, and the evolving validity values of the assignment decisions would result in a “saturation state”. Table 6 in Section 3.3 already mentioned the situations in which the execution of the cluster analysis is interrupted, and the program is terminated with a corresponding return code RC∈{0,−1,−2}:

(1) A valid cluster partitioning was found (RC=0).

(2) No valid cluster partitioning was found, or—using the terminology of circumstantial evidence (see Section 1.2)—the hypotheses formulated in the empirical rules of cluster analysis above could neither be confirmed nor refuted (RC=−1 or RC=−2).

A valid cluster partitioning is obtained when each of the m classification objects is assigned to exactly one of k≤m clusters, with each cluster reference object belonging to the cluster it represents.

The termination criterion is implemented via a function Norm: V→{0, 1}, which normalizes the cluster assignment matrix $D={\left(d_{ij}\right)}_{m,m}\in V^{m\times m}$ after each iteration so that it contains only zeros and ones:

(24) $$Norm(d_{ij})\;=\{\begin{array}{cc} 1 & \;\text{for}\;d_{ij}>e\_UNKNOWN\\ 0 & otherwise \end{array}$$

The actual test of the termination criterion then consists of the following two comparisons:

(25) $$\forall i=1\ldots m:\;1=\sum_{j=1}^{m}Norm\left(d_{ij}\right)$$

(26) $$Norm\left(d_{ij}\right)=1\Rightarrow Norm\left(d_{jj}\right)=1$$

## 4. Conclusions

The examples presented in this paper indicate that empirical logic (EL) provides a flexible and interpretable framework for rule-based decision-making under vague or uncertain conditions. The applications from control engineering and cluster analysis suggest that EL supports intuitive model construction while maintaining a consistent internal structure. In both domains, the resulting rule systems remain comparatively compact and transparent, which facilitates both implementation and interpretation.

The bio-inspired architecture of EL combines explicit knowledge representation with collective rule interaction. Several characteristics distinguish the approach from established soft computing methods:

1. In contrast to artificial neural networks, EL represents knowledge explicitly through rules and categories rather than implicitly encoded in network weights. This structure improves transparency and interpretability.
2. The EL concept of a *category* extends beyond the conventional notion of a fuzzy set. In particular, *free categories* allow vague or uncertain situations to be represented without requiring a predefined reference magnitude (e.g., assignment decisions in cluster analysis).
3. A bipolar validity interval from −1 to +1 prevents conclusions from assigning positive validity simultaneously to a hypothesis and its negation within the EL formalism.
4. In EL-based control applications, the inverse forms of the standardized validity functions allow correcting variables to be determined directly. As a result, the conventional sequence of fuzzification, rule evaluation, and defuzzification used in fuzzy control is not required, thereby preserving a continuous representation of information throughout the control process.
5. Unlike sequential rule-based inference systems, EL relies on the collective interaction of rules. In this respect, the approach is conceptually related to principles known from biological neural networks and associative computational models such as those described by Tank and Hopfield.

The DC drive speed control example suggests that EL can be applied successfully to dynamic control systems (see Section 2). The simulations indicate stable and balanced system behavior with comparatively low sensitivity to parameter variations, despite the use of only a small number of intuitively specified rules. These observations point to potential advantages in applications where interpretability, robustness, and simple model design are important.

The clustering example demonstrates that EL can also be used to address strong heterogeneity in survey populations (see Section 3). By partitioning the data into clusters with high intragroup homogeneity and high intergroup heterogeneity, it provides a basis for statistical analyses [21] (p. 490).

Beyond this application, the discussion introduces an alternative clustering approach that differs substantially from classical hierarchical and partitioning methods:

- Instead of predefined similarity measures, EL uses the category “small distance” represented by the limitation function e_LIMITATION and linked directly to object features. A conjunctive operation via *e_AND* allows multiple bound categories when several features are involved. In practice, both numerical and nominal (especially dichotomous) features can be included without additional coding steps [21] (p. 552).
- Instead of a fusion algorithm, EL employs a dynamic self-organization process that converges to a stationary equilibrium of attractive forces defined by EL rules. The driving force is a free category called “great attractive force”, derived from validity values of objects at “small distance” from a candidate cluster reference object. Cluster granularity is controlled by the parameter CARC (Cluster Attraction Range Coefficient). However, a monotonic sequence of CARC values does not necessarily lead to monotonic refinement or coarsening of clusters, since the method does not construct a hierarchy step by step. Each clustering result is computed independently for a given CARC value. As a consequence, dendrogram-based representations are generally not meaningful or only coincidentally consistent with EL results [27].

Unlike classical methods such as k-means [28] or hierarchical clustering [29], the proposed approach does not optimize an explicit objective function. Instead, cluster structures emerge from the collective interaction of EL rules.

A key aspect of the present work is its focus on practical applicability. In contrast to earlier publications on the theoretical foundations of EL, this paper illustrates how the method can be implemented in concrete application domains. Publicly available software realizations, including Maple prototypes and a Python framework, support direct experimentation and lower the barrier to practical use in research and engineering contexts.

This study is intentionally application-oriented rather than a full evaluation of the method. Systematic benchmarking against established approaches and large-scale analyses is beyond its scope but remains an important topic for future research. Further work may also address larger systems with many interacting rules and potential integrations of EL with machine learning methods.

By combining illustrative applications with executable realizations, this paper lowers the entry barrier to empirical logic and supports its further exploration in applied soft computing.

## Figures and Tables

**Table 1:** Implementation of the EL operators (among various alternatives, the Einstein sum/product (S-/T-norm) and generalized mean (C-/D-norm) operators were found to be particularly effective in an extensive series of comparative experiments (see [12] (p. 32), [2] (pp. 201–218), [13]).

EL Operator	Implementation Function	
e_VALIDATE	*Einstein sum* (S-norm)	$s\left(x,y\right)=\frac{x+y}{1+x\cdot y}$
e_INVALIDATE	*Einstein product* (T-norm)	$t\left(x,y\right)=\frac{x\cdot y}{1+\left(1-x\right)\cdot \left(1-y\right)}$
e_AND	*Generalized mean* (C-norm)	$y={\left(\frac{1}{n}\sum_{i=1}^{n}{x_{i}}^{e\_P}\right)}^{\frac{1}{e\_P}}$ with e_P=−0.5
e_OR	*Generalized mean* (D-norm)	$y={1-\left(\frac{1}{n}\sum_{i=1}^{n}{\left(1-x_{i}\right)}^{e\_P}\right)}^{\frac{1}{e\_P}}$ with e_P=−0.5

**Table 2:** Validity functions of the categories and their arguments (*Validity Threshold* or *Validity Limitation* is the location parameter of the validity function *e_THRESHOLD* or *e_LIMITATION*, defining the position of the zero crossing on the scale of the reference magnitude; *Fuzziness* is a shape parameter derived from the slope at the zero-crossing point of the respective validity function; A justification for the selected values is provided in Section 2.3).

Category Name	Validity Function			
	Operator	Arguments		
		Reference Magnitude	Validity Threshold/ Limitation	Fuzziness
*Controlled_variable_deviation_is_positive*	*e_THRESHOLD*	*e(t)*	0	0.37
*Controlled_variable_deviation_is_negative*	*e_LIMITATION*	*e(t)*	0	0.37
*Change_of_the_controlled_variable_deviation_is_positive*	*e_THRESHOLD*	*c(t)*	0	8
*Change_of_the_controlled_variable_deviation_is_negative*	*e_LIMITATION*	*c(t)*	0	8
*Acceleration_of_the_controlled_variable_deviation_is_positive*	*e_THRESHOLD*	*a(t)*	0	300
*Acceleration_of_the_controlled_variable_deviation_is_negative*	*e_LIMITATION*	*a(t)*	0	300
*Correcting_variable_is_positive*	*e_THRESHOLD*	*u(t)*	0	2.6
*Correcting_variable_is_negative*	*e_LIMITATION*	*u(t)*	0	2.6

**Table 3:** The EL rules of the DC drive control.

*Rule 1*	*IF*		*the controlled variable deviation is positive*
		*OR*	*the change of the controlled variable deviation is positive*
		*OR*	*the acceleration of the controlled variable deviation is positive,*
	*THEN*		*increase the correcting variable.*
*Rule 2*	*IF*		*the controlled variable deviation is negative*
		*OR*	*the change of the controlled variable deviation is negative*
		*OR*	*the acceleration of the controlled variable deviation is negative,*
	*THEN*		*decrease the correcting variable.*

**Table 4:** Determination of the EL controller parameters.

Reference Magnitude	Value Range			Assigned Fuzziness
	Min	Max	Span (Max–Min)	
Controlled variable deviation e(t)	−0.47	1.0	1.47	0.37
Change of the controlled variable deviation c(t)	−21.57	10.39	31.96	8
Acceleration of the controlled variable deviation a(t)	−702.3	470.21	1172.51	300
Correcting variable u(t)	−1.56	3.68	5.24	2.6

**Table 5:** Data set of the application example.

No.	Eurozone Countries (EU-19)	National Debt in % of GDP 2018	Unemployment Rate in % May 2018
1	Belgium	101.50	6.00
2	Germany	60.20	3.40
3	Estonia	8.80	5.00
4	Finland	60.40	7.90
5	France	96.40	9.20
6	Greece	177.80	20.10
7	Ireland	65.60	5.30
8	Italy	130.70	10.70
9	Latvia	37.00	7.40
10	Lithuania	36.00	6.80
11	Luxembourg	22.60	5.20
12	Malta	47.10	3.90
13	Netherlands	53.50	3.90
14	Austria	74.80	4.60
15	Portugal	122.50	7.30
16	Slovakia	49.00	6.80
17	Slovenia	69.30	5.60
18	Spain	97.60	15.80
19	Cyprus	105.70	8.40

**Table 6:** Return codes of the Maple program ExecuteClusterAnalysis.

RC	Meaning
0	Regular termination of program execution with a valid cluster partitioning
−1	Abnormal termination of program execution without a valid cluster partitioning after reaching the specified maximum number of rule iterations
−2	Abnormal termination of program execution without a valid cluster partitioning if no significant change in the cluster assignment matrix can be detected upon further repetition of the rule executions

**Table 7:** EL rules for the diagonal elements of the assignment preference matrix.

*Validation rule* (i=j)	*IF*		*object* i *has a great attractive force as a cluster reference object*
		*AND*	*object* i *is not already assigned to another cluster* k
	*THEN*		*validate the decision to select object* i *as a cluster reference object.*
*Invalidation rule* (i=j)	*IF*		*object* i *does not have a great attractive force as a cluster reference object*
		*OR*	*object* i *is already assigned to another cluster* k
	*THEN*		*invalidate the decision to select object* i *as a cluster reference object.*

**Table 8:** EL rules for the off-diagonal elements of the assignment preference matrix.

*Validation rule* (i≠j)	*IF*		*object* i *and object* j *are similar*
		*AND*	*object* j *is a cluster reference object*
		*AND*	*object* i *is not already assigned to another cluster* k
	*THEN*		*validate the decision that object* i *is assigned to cluster* j.
*Invalidation rule* (i≠j)	*IF*		*object* i *and object* j *are not similar*
		*OR*	*object* j *is not a cluster reference object*
		*OR*	*object* i *is already assigned to another cluster* k
	*THEN*		*invalidate the decision that object* i *is assigned to cluster* j.

## Data Availability

The Maple prototypes and test results described in this article are available in the EU open research repository Zenodo [3] (https://zenodo.org/records/14962362, accessed on 3/3/2025). Empirical Logic (version 1.0.1) is available as a Python framework for general use [4] (https://doi.org/10.5281/ZENODO.14962362).
